# Cardiovascular manifestations in severe and critical patients with COVID‐19

**DOI:** 10.1002/clc.23384

**Published:** 2020-06-20

**Authors:** Qingxing Chen, Lili Xu, Yongbin Dai, Yunlong Ling, Jiahao Mao, Juying Qian, Wenqing Zhu, Wencheng Di, Junbo Ge

**Affiliations:** ^1^ Department of Cardiology Zhongshan Hospital, Fudan University, Shanghai Institute of Cardiovascular Diseases Shanghai China; ^2^ Department of Cardiology The Third People's Hospital of Shenzhen Shenzhen China; ^3^ Department of Cardiology People's Hospital of Cixi, Wenzhou Medical University, Cixi Zhejiang China

**Keywords:** cardiac injury, cardiovascular manifestations, COVID‐19, critical status, hypotension

## Abstract

**Background:**

Severe acute respiratory syndrome coronavirus 2 (SARS‐CoV‐2) could cause virulent infection leading to Corona Virus Disease 2019 (COVID‐19)‐related pneumonia as well as multiple organ injuries.

**Hypothesis:**

COVID‐19 infection may result in cardiovascular manifestations leading to worse clinical outcome.

**Methods:**

Fifty four severe and critical patients with confirmed COVID‐19 were enrolled. Risk factors predicting the severity of COVID‐19 were analyzed.

**Results:**

Of the 54 patients (56.1 ± 13.5 years old, 66.7% male) with COVID‐19, 39 were diagnosed as severe and 15 as critical cases. The occurrence of diabetes, the level of D‐dimer, inflammatory and cardiac markers in critical cases were significantly higher. Troponin I (TnI) elevation occurred in 42.6% of all the severe and critical patients. Three patients experienced hypotension at admission and were all diagnosed as critical cases consequently. Hypotension was found in one severe case and seven critical cases during hospitalization. Sinus tachycardia is the most common type of arrythmia and was observed in 23 severe patients and all the critical patients. Atrioventricular block and ventricular tachycardia were observed in critical patients at end stage while bradycardia and atrial fibrillation were less common. Mild pericardial effusion was observed in one severe case and five critical cases. Three critical cases suffered new onset of heart failure. Hypotension during treatment, severe myocardial injury and pericardial effusion were independent risk factors predicting the critical status of COVID‐19 infection.

**Conclusion:**

This study has systemically observed the impact of COVID‐19 on cardiovascular system, including myocardial injury, blood pressure, arrythmia and cardiac function in severe and critical cases. Monitoring of vital signs and cardiac function of COVID‐19 patients and applying potential interventions especially for those with hypotension during treatment, severe myocardial injury or pericardial effusion, is of vital importance.

**Abbreviations**
ACEI: angiotensin converting enzyme inhibitor
ARB: angiotensin II receptor blocker
AVB: atrioventricular block
CCB: calcium channel blocker
CI: confidence interval
CK‐MB: creatinine kinase‐MB isoenzyme
COVID‐19: Corona Virus Disease 2019
CRP: C reactive protein
ECG: electrocardiogram
ESC: European Society of Cardiology
ESH: European Society of Hypertension
ESR: erythrocyte sedimentation rate
IL‐6: interleukin‐6
NT‐proBNP: N‐terminal pro‐B‐type natriuretic peptide
OR: odds ratio
SARS‐CoV‐2: severe acute respiratory syndrome coronavirus 2
TnI: troponin I
WBC: white blood cell

## 1 INTRODUCTION

Corona Virus Disease 2019 (COVID‐19), an outbreak of pneumonia caused by severe acute respiratory syndrome coronavirus 2 (SARS‐CoV‐2) in Wuhan in December 2019, has spread rapidly throughout China and to other regions of the world, including Europe, the United States, Japan and Australia.^1^ COVID‐19 is a contagious disease that affects multiple organs of the body and reported complications included pneumonia, derangement of renal and liver functions, cardiac injuries, cellular immune deficiency and coagulation activation.^2^, ^3^, ^4^ It is reported that about 12% of patients with COVID‐19 suffered cardiac injury.^5^ However, cardiovascular manifestations after COVID‐19 infection has not been reported systemically. This study was aimed to explore how COVID‐19 affects cardiovascular system including myocardium, conduction systems, cardiac function and blood pressure especially in severe and critical cases and to identify potential risk factors predicting the severity of COVID‐19.

## 2 METHODS

### 2.1 Data collection

We collected medical records of 54 patients diagnosed as severe and critical cases admitted to hospital from January 27 to February 28, 2020. All the patients enrolled in this study were diagnosed according to the sixth edition guideline issued by China's National Health Commission. Severe cases were classified when meeting any one of the following: (1) respiratory distress: RR > =30times/min; (2) fingertip oxygen saturation < =93% under resting state; (3) arterial partial pressure of oxygen (PaO_2_)/fraction of inspiration O_2_ (FiO_2_) < =300 mmHg (1 mmHg = 0.133 kPa). Critical cases were classified when meeting any one of the following: (1) respiratory failure and an artificial airway required for invasive mechanical ventilation; (2) shock; (3) combining failure of other organs which requires ICU monitoring and treatment. Clinical, laboratory, electrocardiogram (ECG) and echocardiographic data were obtained from electronic medical record system and reviewed by a trained team of cardiologists. This study was approved by the institutional ethics.

### 2.2 Cardiovascular assessment

Blood pressure and pulse rate were measured at least every 4 hours during hospitalization using automatic blood pressure recording machines. Hypotension was defined as systolic blood pressure <90 mmHg and/or diastolic blood pressure <60 mmHg. Tachycardia was defined as a heart rate >100 beats/minute in at least two consecutive readings. Bradycardia was defined as a daytime heart rate <50 beats/minutes while awake in at least two consecutive readings. The temperature was charted every 4 hours by eardrum thermometers. Patients were checked for new onset or change of symptoms. Blood tests were performed regularly. Cardiac injury was defined if the serum level of specific cardiac biomarkers (eg, troponin I) was above 99% upper reference limit. Serious cardiac injury was defined if the serum level of troponin I (TnI) was above 3 times upper reference limit.^6^ Echocardiography was performed when it was deemed necessary by the physician in charge, such as significant hypotension that necessitated inotropic support or when there were suspicious changes of myocardial ischemia on electrocardiogram. ECG was performed when clinically indicated, such as the occurrence of unexplained or severe tachycardia, bradycardia, or hypotension, or when cardiac arrhythmia was suspected.

### 2.3 Statistical analysis

Continuous variables were expressed as the means and SDs. Categorical variables were summarized as the counts and percentages in each category. For comparison of non‐parametric variables, Pearson *χ* ^2^ test was used. Multi‐factor logistic regression model was used to analyze the related factors of COVID‐19 critical type. A *P* value <.05 was considered significant.

## 3 RESULTS

### 3.1 Demographic and clinical characteristics

Among 54 patients enrolled in our study, 39 (72.2%) were diagnosed as severe and 15 (27.8%) as critical cases. Thirty‐six (66.7%) patients were males. Gender differences were similar in severe and critical groups. The median age was 57.6 (44.9‐70.3, between 25 and 78) years old. Patients over 65 years old occupied almost one third, while the average age was similar in severe and critical groups. Nine patients had a history of cardiocerebrovascular diseases, including six patients with coronary artery disease (four severe and two critical cases), one patient with aortic and mitral valve replacement (severe case), one patient with mild to moderate mitral regurgitation (critical case) and one patient with persistent atrial fibrillation and acute cerebral infarction (critical case). None of the cases studied had a previous history of chronic heart failure or chronic lung disease. The presenting symptoms of fever occurred in 47 (87.0%) patients, including 34 (63.0%) low‐grade fever, 9 (16.6%) moderate fever and 4 (7.4%) high fever, respectively. No difference in fever type was observed between severe and critical group. The occurrence of diabetes and the level of D‐dimer, white blood cell (WBC), procalcitonin, interleukin‐6 (IL‐6), erythrocyte sedimentation rate (ESR) and C‐reactive protein (CRP) in critical cases were significantly higher than severe cases (*P* < .01), indicating that patients with diabetes and a higher level of D‐dimer and inflammation are more susceptible to develop critical conditions. Cardiac biomarkers were remarkedly higher in critical group (*P* < 0.01), suggesting more serious myocardial injury. Renal function biomarkers were similar in severe and critical cases, while acute kidney injury occurred in about one third of patients in both groups. Clinical features and laboratory findings were summarized in Table 1.

**TABLE 1:** Demographic and clinical characteristics in severe and critical patients

	Severe (N = 39)	Critical (N = 15)	*P* value
Gender (male)	27 (69.2%)	9 (60%)	.536
Age (years)	56.1 ± 13.5	61.7 ± 9.6	.15
Age > 65	10 (25.6%)	7 (46.7%)	.192
Hypertension	11 (28.2%)	5 (33.3%)	.714
Diabetes	**13 (33.3%)**	**12 (80%)**	**<.01**
Coronary artery disease	4 (10.3%)	2 (13.3%)	.75
History of PCI	2 (5.1%)	0 (0%)	.518
Chronic heart failure	0 (0%)	0 (0%)	—
Chronic lung disease	0 (0%)	0 (0%)	—
Symptoms			
Chest pain	2 (5.1%)	2 (13.3%)	.306
Myalgia	5 (12.8%)	5 (33.3%)	.110
Fever	34 (87.2%)	13 (86.7%)	.95
Low‐grade fever	26 (66.7%)	8 (53.3%)	.745
Moderate fever	6 (15.4%)	3 (20%)	.682
High fever	2 (5.1%)	2 (13.3%)	.306
Laboratory findings			
WBC (*10^9/L)	**6.44 ± 2.64**	**11.24 ± 9.07**	**<.01**
Markedly elevated WBC (*10^9/L)	**0 (0%)**	**2 (13.3%)**	**<.01**
Procalcitonin (μg/L)	**0.080 ± 0.010**	**1.56 ± 0.85**	**<.01**
IL‐6 (pg/mL)	**25.00 ± 3.90**	**83.31 ± 29.78**	**<.01**
ESR (mm/H)	**51.42 ± 3.68**	**70.33 ± 8.88**	**.023**
CRP (mg/L)	**40.89 ± 6.32**	**111.37 ± 14.18**	**<.01**
Hemoglobin (g/L)	130.9 ± 25.6	118.1 ± 23.6	.11
D‐dimer (mg/L)	**1.18 ± 1.95**	**3.12 ± 2.99**	**<.01**
BUN (mmol/L)	6.92 ± 7.79	6.77 ± 3.31	.94
Scr (μmol/L)	75.2 ± 21.9	83.5 ± 45.3	.37
eGFR (mL/min/1.73m^2^)	92.2 ± 19.8	82.9 ± 31.7	.21
Acute kidney injury	13 (33.3%)	5 (33.3%)	.95

*p* < 0.05 means statistically significant

### 3.2 Cardiac injury in severe and critical cases

Four patients (two severe and two critical) complained of chest pain, but none of them were found to have TnI elevation or ECG change. Presence of chest pain may be due to pleuritis caused by pneumonia. Elevation of TnI was observed in 23 (42.6%) patients. Notably three patients with highly elevated TnI (>3 times the upper reference limit), indicative of severe myocardial injury, were all critical cases. The serum levels of TnI, CK‐MB and myoglobin were significantly higher in critical group compared to severe group (*P* < .01). In addition, elevation of TnI was consistent with the elevation of inflammatory markers especially procalcitonin and IL‐6 (Supporting Information Figure S1), indicating the role of inflammation and cytokine storm in cardiac injury. ST‐T changes in ECG were identified in four patients (one severe and three critical) and three of them were accompanied by increase of TnI. NT‐proBNP elevation occurred in 30 (55.6%) patients. Eight patients presented with significantly elevated NT‐proBNP (>3 times the upper reference limit), including five critical cases. The concentration of NT‐proBNP were remarkedly higher in critical group, compared to severe group (*P* < .01) (Table 2).

**TABLE 2:** Cardiac injury in severe and critical patients

	Severe (N = 39)	Critical (N = 15)	*P* value
LDH (U/L)	512.7 ± 350	792 ± 608	.332
CK‐MB (ng/mL)	**0.71 ± 0.58**	**3.07 ± 3.92**	**<.01**
TnI (ng/mL)	**0.014 ± 0.012**	**0.29 ± 0.68**	**.01**
Severe myocardial injury	**0 (0%)**	**3 (20%)**	**<.01**
Myoglobin (ng/mL)	**103 ± 7**	**489 ± 814**	**<.01**
NT‐proBNP (pg/mL)	**341.5 ± 435.5**	**1582 ± 2374**	**<.01**
ST‐T change in ECG	1 (2.6%)	3 (20%)	.3

*p* < 0.05 means statistically significant

### 3.3 Hypertension and hypotension in severe and critical cases

Sixteen (29.6%) patients had a history of hypertension in this study. No significant difference was found in the percentage of hypertension between severe and critical patients. Among 16 patients with hypertension, 8 were taking calcium channel blockers (CCBs), 3 were taking angiotensin converting enzyme inhibitors (ACEIs)/ angiotensin II receptor blockers (ARBs), 3 were taking β‐blockers and 5 were taking antihypertensive drugs irregularly. None of them were taking diuretics. Nine patients with a history of hypertension and seven patients without a history of hypertension suffered from high blood pressure at admission. All these patients were managed after admission according to 2018 ESC/ESH Guidelines for the Management of Arterial Hypertension. Three patients experienced hypotension at admission and all of them were diagnosed as critical cases consequently. During treatment hypotension was found in 1 (2.6%) severe case and 7 (46.7%) critical cases (*P* < .01), including 3 ended up with death. Only one patient suffered from transient hypotension and recovered later. These data suggested that critical patients are prone to present with persistent refractory shock in the course of COVID‐19 infection, a sign of poor prognosis (Table 3).

**TABLE 3:** Cardiac‐related clinical characteristic in severe and critical patients

	Severe (N = 39)	Critical (N = 15)	*P* value
Blood pressure			
History of hypertension	11 (28.2%)	5 (33.3%)	.714
Hypertension at admission	10 (25.6%)	6 (40%)	.308
Hypotension at admission	**0 (0%)**	**3 (20%)**	**<.01**
Hypotension during treatment	**1 (2.6%)**	**7 (46.7%)**	**<.01**
Electrocardiogram			
Sinus tachycardia	**23 (59.0%)**	**15 (100%)**	**<.01**
Premature beat	8 (20.5%)	2 (13.3%)	.547
Ventricular tachycardia	1 (2.6%)	2 (13.3%)	.125
Atrioventricular block	**0 (0%)**	**2 (13.3%)**	**.02**
Sinus bradycardia	2 (5.1%)	1 (6.7%)	.825
Atrial fibrillation	0 (0%)	1 (6.7%)	.278
Echocardiography			
Pericardial effusion	**1 (2.6%)**	**5 (33.3%)**	**<.01**
New onset of heart failure	**0 (0%)**	**3 (20%)**	**<.01**
Left heart failure	0 (0%)	1 (6.7%)	.278
Right heart failure	**0 (0%)**	**2 (13.3%)**	**.02**

*p* < 0.05 means statistically significant

### 3.4 Cardiac arrythmia in severe and critical cases

Sinus tachycardia is the most common type of arrythmia and was observed in 23 (59.0%) severe patients and all the critical patients (*P* < .01). It is noteworthy that the rising of heart rate was not consistent with body temperature and oxygen saturation. The heart rate remained on a high level even during the rehabilitative phase. No significant difference of premature beat was found between severe and critical group. Only one severe patient experienced transient ventricular tachycardia during hospitalization while another two critical patients suffered from persistent refractory ventricular tachycardia and unfortunately ended up with death. Atrioventricular block (AVB) was seen in two critical patients, one first‐degree AVB and the other Mobitz type I second‐degree AVB. Three patients experienced sinus bradycardia, including two severe cases with a previous history of bradycardia and one critical case at end stage. Atrial fibrillation was rare and only one critical patient presented with paroxysmal atrial fibrillation (Table 3).

### 3.5 Echocardiographic findings in severe and critical cases

We performed echocardiography in 31 patients, including 13 severe and 18 critical patients. Occurrence of mild pericardial effusion was observed in one severe case and five critical cases (*P* < .01), suggesting critical patients are more likely to develop pericardial effusion. Three critical cases suffered from new onset of heart failure. Two cases presented with right heart failure, accompanied by right heart enlargement and pulmonary hypertension and unfortunately ended up with death. One case presented with reversible left heart failure and elevation of TnI, indicative of possibility of myocarditis, but it is a pity that we failed to obtain the histopathology. During hospitalization, cardiomegaly was not found in any patient without heart failure (Table 3).

### 3.6 Factors predicting the critical status of COVID‐19 infection

Multifactor logistic regression analysis showed that severe cardiac injury (OR = 2.4, 95%CI 1.8‐20.1, *P* = .04), hypotension during treatment (OR = 3.4, 95%CI 2.1‐17.1, *P* = .04) and pericardial effusion (OR = 3.5, 95%CI 1.8‐15.1, *P* = .05) were independent risk factors predicting the critical status of COVID‐19 infection (Table 4).

**TABLE 4:** Multi‐factor logistic regression analysis of factors predicting the critical status of COVID‐19 infection

	OR	95% CI	*P* value
Diabetes	2.165	0.036‐1.608	.141
Hypotension during treatment	**3.462**	**2.086‐17.146**	**.043**
Mild pericardial effusion	**3.430**	**1.803‐15.180**	**.047**
CK‐MB	0.785	0.549‐4.897	.376
D‐dimer	0.525	0.789‐1.675	.469
Severe cardiac injury	**2.421**	**1.812‐20.112**	**.044**

*p* < 0.05 means statistically significant

## 4 DISCUSSION

This study focused on cardiovascular manifestations and clinical risk factors in severe and critical patients infected with SARS‐CoV‐2. It is the first time to systemically report cardiovascular manifestations after COVID‐19 infection. The average age of our study population was 57.6 ± 12.7 years old and aged people older than 65 years accounted for one‐third. Nearly two‐third of the patients were male in whether severe or critical cases. The age and gender distribution in this study were consistent with the data recently published in Lancet,^4^ suggesting that old adult male patients are more likely to develop critical conditions and deserve more attention. We suspected that fewer females in severe and critical cases may partly owe to the protection of X chromosome and estrogens in female patients. Another study from Wuhan^7^ reported that diabetic patients occupied 22.22% of the critical cases infected with SARS‐CoV‐2 and this percentage was significantly higher than non‐critical cases. The proportion of diabetic patients in critical cases was even higher in our study (80%), urging us to pay more attention to the influence of diabetes in the progression and deterioration of COVID‐19.

It is reported that about 12% of patients with COVID‐19 suffered cardiac injury in previous studies.^7^, ^8^ This percentage is significantly higher in this study partly because the patients enrolled were severe and critical cases, which is different from study population in other studies. TnI elevation occurred in 42.6% of patients while NT‐proBNP elevation occurred in 55.6% of patients. Among these patients most presented with mild elevation of cardiac markers while a few presented with significant elevation. This result urged us to pay special attention to cardiac injury and heart protection in patients with COVID‐19, especially those in severe and critical status. The mechanism of acute myocardial injury caused by SARS‐CoV‐2 infection might be related to cardiomyocyte damage and apoptosis due to infiltration of inflammatory cells, cytokine storm, hypoxemia, acidosis, pulmonary hypertension, increased myocardial oxygen consumption and even mental stress. Significantly higher D‐dimer level in critical cases suggested a possible role of microembolism in causing cardiac injury. Other proposed mechanisms of myocardial injury included ACE2‐mediated direct damage of cardiomyocyte, plaque instability in patients with previous coronary artery disease and fulminant myocarditis accompanied with pneumonia.

In our study blood pressure in 56% patients with a history of hypertension were out of control at admission. It may be attributed to increased catecholamine related to stress and anxiety and ACE2 pathway influenced by SARS‐CoV‐2 infection.^9^ It is noteworthy that three patients suffered from hypotension at admission and seven patients suffered from persistent hypotension during hospitalization and all of them developed critical status consequently. Presence of hypotension in patients with COVID‐19 was different from that in SARS. We considered the main reason of hypotension was septic shock resulting from severe infection. Other reversible causes included inadequate intake, fever and perspiration, leading to hypovolemia and electrolyte imbalances. Hypotension was an independent risk factor for critical status and a poor prognosis, therefore blood pressure should be closely monitored during the hospitalization period and further attention should be paid to those developing hypotension.

Cardiac arrythmia in patients with COVID‐19 was similar to patients with SARS in 2003.^10^ Sinus tachycardia was the most common type of arrythmia in patients with COVID‐19, especially in severe and critical cases. Persistent tachycardia was not consistent with body temperature and oxygen saturation. The heart rate remained on a high level even during the rehabilitative phase, probably due to long lasting change in the autonomic tone, cardiopulmonary or peripheral deconditioning and myocardial injury. Ventricular tachycardia and atrioventricular block were not common and mainly occurred in critical status and end stage of the disease. They could act as warning signs for disease deterioration. In addition, atrial fibrillation was rare in severe and critical patients with COVID‐19. To sum up, close observation of vital signs and regular ECG test is strongly advised during treatment.

Echocardiographic data showed occurrence of mild pericardial effusion in about one‐third of critical patients without manifestation of heart failure, which might be due to inflammatory effusion. Three critical cases were reported to suffer from new onset of heart failure: two cases developed right heart failure after “white lung” at end stage of the disease; one case presented with reversible left heart failure and TnI elevation, indicative of occurrence of myocarditis but regrettably lack of pathological evidence. In other patients without heart failure, no impaired diastolic function, abnormal wall motion or cardiomegaly were found, which was inconsistent with previous publication.^10^ More detailed studies are needed to get a comprehensive understanding of cardiovascular manifestations and cardiac function change of SARS‐CoV‐2 infection.

### 4.1 Clinical implications

It is the first time to systemically report cardiovascular manifestations after COVID‐19 infection, providing some reference for clinical practice. Our results suggested that more attention should be paid to old adult male patients and diabetic patients who are more susceptible to develop critical conditions. The occurrence of cardiac injury in our study is significantly higher than data in previous studies probably due to different severity of enrolled patients, urging us to attach importance to heart protection when choosing optimal treatment strategies especially in managing those severe and critical cases. Hypotension was an independent risk factor for critical status and poor prognosis. Therefore, blood pressure should be closely monitored during the hospitalization period. Sinus tachycardia was the most common type of arrythmia in patients with COVID‐19, especially in severe and critical cases. Ventricular tachycardia and atrioventricular block were less common, but they may act as warning signs for disease deterioration. Close observation of vital signs is advised. Mild pericardial effusion occurred in about one‐third of critical patients without manifestation of heart failure and was another independent risk factor for critical disease status. More detailed studies are needed to get a deeper understanding of cardiovascular manifestations of SARS‐CoV‐2 infection.

### 4.2 Limitations

Main limitation of this study was small sample size. Only 54 confirmed severe and critical COVID‐19 patients from single center were enrolled, making it difficult to get a full understanding of the relation between COVID‐19 and cardiovascular system. Besides, the follow‐up data should be collected to get a knowledge of the long‐term impact of COVID‐19 on cardiovascular system.

## 5 CONCLUSIONS

This study has systemically reported the impact of COVID‐19 on cardiovascular system, including myocardial injury, blood pressure, arrythmia and cardiac function in severe and critical cases. Hypotension during treatment, severe myocardial injury and pericardial effusion were the independent risk factors of critical disease status in patients with COVID‐19. Monitoring of vital signs and cardiac function of COVID‐19 patients and applying potential interventions especially for those with hypotension during treatment, severe myocardial injury or pericardial effusion, is of vital importance.

## Supporting information


**Figure S1** Inflammatory markers in severe and critical patients.
