# Supplementary figures and images for: Cardiovascular manifestations in severe and critical patients with COVID‐19
Source: Clin Cardiol. 2020 Jun 20;43(7):796–802. doi: 10.1002/clc.23384 (PMC7323347; doi:10.1002/clc.23384)

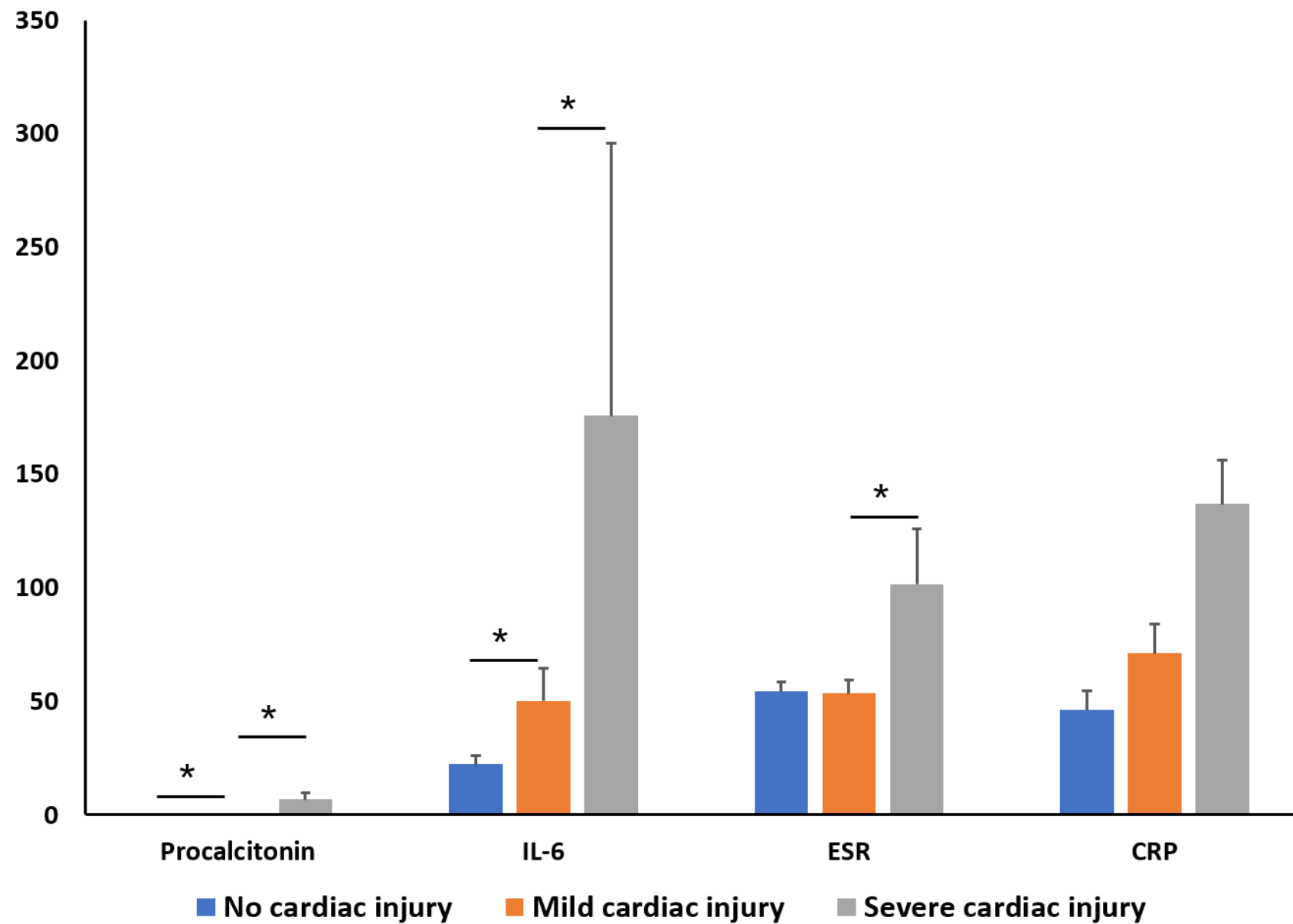

Supplement: Supplementary file 1 — Figure S1 Inflammatory markers in severe and critical patients. [file CLC-43-796-s001.pdf]
